# Review of the factors influencing the motivation of community drug distributors towards the control and elimination of neglected tropical diseases (NTDs)

**DOI:** 10.1371/journal.pntd.0006065

**Published:** 2017-12-06

**Authors:** Alison Krentel, Margaret Gyapong, Shruti Mallya, Nana Yaa Boadu, Mary Amuyunzu-Nyamongo, Mariana Stephens, Deborah A. McFarland

**Affiliations:** 1 Bruyere Research Institute, Ottawa Canada; 2 Institute for Health Research, University of Health and Allied Sciences, Ho Ghana; 3 Health and Nutrition Bureau, Global Affairs Canada, Ottawa Canada; 4 African Institute for Health and Development, Nairobi Kenya; 5 NTD Support Center, Task Force for Global Health, Decatur GA United States of America; 6 Rollins School of Public Health, Emory University Atlanta GA United States of America; Brown University, UNITED STATES

## Abstract

**Background:**

Community drug distributors or neglected tropical disease (NTD) volunteers have played a crucial role in ensuring the success of mass drug administration (MDA) programs using preventive chemotherapy (PC) for lymphatic filariasis, onchocerciasis, schistosomiasis, trachoma and soil transmitted helminths. In recent years however, a noticeable decline in motivation of some of these volunteers has been perceived, potentially negatively impacting the success of these programs. Potential hypotheses for this change in motivation include the long duration of many MDA programs, the change in sociocultural environments as well as the changes to the programs over time. This literature review identifies factors that affect NTD volunteer performance and motivation, which may be used to influence and improve future programming.

**Methodology/Principal findings:**

A systematic search was conducted to identify studies published between January 1995 and September 2016 that investigate factors pertaining to volunteer motivation and performance in NTD drug distribution programs. Searches from several databases and grey literature yielded 400 records, of which 28 articles from 10 countries met the inclusion criteria. Quality assessment of studies was performed using the Critical Appraisal Skills Programme(CASP) checklist. Data pertaining to motivation, performance, retention and satisfaction was extracted and examined for themes. Recurring themes in the literature included monetary and material incentives, intrinsic motivation, gender, cost to participate, and health systems and community support. Of these, community support and the health system were found to be particularly impactful. Very few studies were found to explicitly look at novel incentives for volunteers and very few studies have considered the out of pocket and opportunity costs that NTD volunteers bear carrying out their tasks.

**Conclusions/Significance:**

There is currently great interest in incorporating more attractive incentive schemes for NTD volunteers. However, our results show that the important challenges that volunteers face (cultural, health systems, financial and community related) may have less to do with financial incentives and may actually have a larger impact on their motivation than has previously been understood. Further integration of NTD programs into existing health systems is expected to improve the NTD volunteer working environment. Relevant community engagement related to the MDA program should also provide the supportive environment needed in the community to support NTD volunteers. Programs need to consider these issues to improve working conditions for NTD volunteers.

## Author summary

Volunteer community drug distributors play an important role in preventing and treating many neglected tropical diseases (NTD) through their participation in mass drug administration (MDA) programs for lymphatic filariasis (elephantiasis), onchocerciasis (river blindness), soil transmitted helminths (intestinal worms), trachoma and schistosomiasis (snail fever). Their role is to carry out a census, inform and educate community members, distribute medication, fill out reports and monitor for adverse events. These volunteers work tirelessly in their communities with minimal incentives. Increasingly, as the 2020 elimination goals for NTDs approach, there is concern that we need to better motivate these individuals and prevent attrition. In order to better understand what motivates volunteer community drug distributors, a review of the literature was carried out. Recurring themes in the literature included monetary and material incentives, intrinsic motivation, gender, cost to participate, and health systems and community support. Of these, community support and the health system were found to be particularly impactful. Our results show that better community engagement and integration into the health system will help to maximize retention of this volunteer workforce.

## Introduction

Since 1995, the global community has been committed to reducing the impact of neglected tropical diseases on the poorest populations through the administration of preventive chemotherapy (PC) and medical care for people suffering from chronic manifestations of these diseases. Referred to as neglected tropical diseases (NTD), this suite of infectious diseases has historically not received the global attention and funding to match the significant burden of disease they exact in the poorest populations. Since 2012, the international community has agreed to the deadline of 2020 to control, eliminate and eradicate 17 NTDs. These ambitious goals are being supported by a global partnership of public, private, research and academic institutions.The history of the commitment to reducing NTDs began in the 1990s. With the establishment of the African Program for Onchocerciasis Control (APOC) in 1995 and the World Health Assembly resolution 50.29 which committed to eliminate lymphatic filariasis (LF) as a public health problem, global attention increased for these two diseases. The elimination of LF and onchcocerciasis has been facilitated by massive and long-term donations from pharmaceutical companies to provide the drugs required for mass drug administration (MDA) to people living in endemic regions (Mectizan by Merck, Albendazole by GlaxoSmithKline and Diethylcarbamazine citrate by Eisai Pharmaceuticals). As potential for success was achieved in the elimination and control of onchocerciasis and lymphatic filariasis, additional NTDs were slated for control and elimination through preventive chemotherapy, namely schistosomiasis, trachoma and soli-transmitted helminthiases (STH). With that decision, additional drug donations of Mebendazole (Johnson & Johnson), Praziquantel (Merck) and Azithromycin (Pfizer) were secured to support NTD programmes. In 2012, The London Declaration was signed documenting a unique and unprecedented partnership of governments, industry, academia, non-governmental organisations (NGOs) and donors, all united to support the eradication, elimination and control of NTDs in alignment with the World Health Organization (WHO) 2020 Roadmap on NTDs [www.unitingtocombatntds.org].

Significant public health gains have been made and documented in the process to reduce the transmission and impact of PC-NTDs, specifically those NTDs that respond to preventive chemotherapy and include: lymphatic filariasis; schistosomiasis; onchocerciasis; trachoma; and STH. By the end of 2014, APOC reported that 65% of the population living in onchocerciasis endemic regions in Africa had received ivermectin.[1] It is estimated that by the end of 2015, APOC had saved 17.4 million Disability Adjusted Life Years (DALYs) in its 20 years of existence.[2] The Global Program to Eliminate Lymphatic Filariasis (GAELF) was launched in 2000. Between 2000 and 2014, the program delivered 5.6 billion treatments to 763 million people living in endemic regions, saving an estimated 175 million DALYs.[3] The economic gains of reducing LF in the first eight years of the program (2000–2007) are estimated as US $21.9 million in direct economic benefits and approximately US $2.2 billion in savings for health systems due to the decreases in services needed for LF patients[4]. Schistosomiasis control has been successful in a number of countries, however it remains a major contributor to suffering for people living in tropical regions where the disease remains prevalent.[5] The estimated DALYs lost from schistosomiasis is 24–27 million, with the highest burden of disease in Africa.[5]

The public health gains of the NTD platform are noteworthy and the cost of delivering these programs is considered inexpensive, in part because of the role of volunteers. A recent review confirmed that the cost of delivering treatments for six PC-NTDs is estimated to be less than $0.50 USD per person treated, except where programs cannot rely on local volunteers.[6] With all of the noted public health gains that have been demonstrated since the beginning of the global focus on NTDs, it is important to acknowledge the crucial role that local volunteers play in the implementation of these NTD elimination and control programs. Known by different names—community drug distributors (CDDs), NTD volunteers, health extension worker, community health workers (CHWs), health assistants or health army—their role is the same: to ensure that each person eligible to receive PC treatment for NTDs takes the drugs as per the protocols. The participation of these community volunteers reduces the costs of NTD control and elimination as well as provides an effective way to reach endemic communities.

Community drug distributors rose to importance within APOC, when the community directed treatment with ivermectin (CDTI) was initiated.[7] Communities were responsible to select volunteers who would conduct census activities, social mobilization and drug delivery ensuring that they reached all eligible community members. With CDTI, a massive volunteer workforce was mobilised. In 2013 alone, 517,000 CDDs were trained through APOC.[1] Subsequent NTD programs have adopted the use of community volunteers to deliver drugs to their fellow community members under the supervision of the primary health care system and other local organizations.

A recent review by Corley *et al* (2016) remarked on the importance of CDDs in motivating and educating communities for sustainability and uptake of NTD activities. They are considered a “valuable human resource” and program managers should recognise their contribution while acknowledging the risk that these NTD volunteers can also inhibit programmatic achievements.[8] Many reviews of compliance and coverage of MDA programs cite the role that NTD volunteers play, both as positive and negative influences on programmatic outcomes.[9] Within the context of delivering drugs for the MDA for LF, some of the positive ways that NTD volunteers impact coverage and compliance include: being familiar to community members; taking the drugs themselves in front of community members; and visiting a household prior to drug distribution.[9] CDDs can also negatively impact the coverage and compliance with MDA activities for LF elimination by having poor motivation and poor communication skills.[10] In onchocerciasis programs, community perception of the drug distributor has been shown to influence uptake of the pills.[11–13] In a study on schistosomiasis control in Kenya, community members reported that they did not feel comfortable being treated by a non-medical person and by someone who did not have a certificate to demonstrate his/her knowledge or training in the disease and treatment. The CDD has not always been adequately supported by the health system so that s/he can carry out the activities sufficiently, thereby negatively affecting the uptake of praziquantel in this study.[14]

While the critical importance of CDDs is essential to the successes of NTD programs, much of our understanding of their role comes from the earlier days of the programs. The social, cultural and programmatic landscape have changed with potentially consequential effects on the acceptance and performance of the volunteers. NTD programs began as single disease vertical programs (APOC, GPELF) but now have been integrated at the global, national and community levels to maximise delivery and funding platforms. With this integration, the role of communty drug distributors is more signficant as these individuals are now responsible for delivering multiple drug combinations, maintaining different reporting forms and communicating at different intervals throughout the year with their communities. To date, we do not fully understand how this increase of responsibilities has impacted the motivation and retention of these volunteers.

The cultural landscape in many of these communities has also changed, with the increase in mobile telephone technology and urbanization for example. The impact of technological and other socio-cultural changes on the motivation and performance of volunteer community drug distributors has not been fully explored. Do the often-used traditional forms of incentives (t-shirts and badges) work in a more sophisticated environment? Additionally, many communities have now had multiple rounds of MDA, and community fatigue with MDA has been anecdotally reported. Finally, community members are no longer seeing the manifestation of the NTD diseases as they previously saw, thereby potentially reducing the urgency of compliance. How might these changes in the community perception of NTDs and their elimination affect the motivation of the CDDs?

This paper seeks to review in a systematic manner the current literature examining the motivation, retention and performance of CDDs for five PC diseases (trachoma, onchocerciasis, LF, STH and schistosomiasis). For the purposes of this paper and due to the many different names used for these individuals, all CDDs will be referred to as NTD volunteers.

## Methods

### Search strategy

A comprehensive search for peer-reviewed and non-peer-reviewed literature was performed in September 2016. Prior to searching databases, the appropriateness of search terms was verified for identifying relevant literature. Specifically with regards to the NTD volunteer: *community drug distributor(s)*, *community health worker(s)*, *community health volunteer(s)*. Terms for the five NTDs for which preventive chemotherapy treatment (PCT) is available, were reviewed including: *onchocerciasis* or *river blindness*, *trachoma*, *schistosomiasis* or *bilharzia*, or *soil-transmitted helminth infections* and *lymphatic filariasis or elephantiasis*. This was done by comparing the selected terms to controlled vocabulary terms such as Medical Subject Headings (MeSH) terms and key index terms from known or retrieved key references on the use of CDDs in global NTD programs.

A search strategy comprising a combination of the verified search terms with key phrases such as ‘incentive(s)’, ‘motivation’, ‘performance’, and ‘satisfaction’ was applied to the following bibliographic databases: PubMed/MEDLINE; Global Health; EMBASE; CINAHL; Africa-Wide Information; ELDIS; APPI-Centre; IBSS; Open Grey; and Web of Science. A manual search of references from retrieved articles, databases, and stakeholder websites including the CDC, The London Centre for Neglected Tropical Disease Research, The NTD Modelling Consortium, COR-NTD, Global Network of Neglected Tropical Diseases, The End Fund, and the WHO, to identify relevant, non-peer reviewed (grey) literature was further conducted. For completeness, manual searching also included the reference lists of recent issues from relevant journals including PLOS Neglected Tropical Diseases. The search strategy cast a wide net to capture pertinent literature on CDD participation in global PCT programs focused on the five selected NTDs, including all identified synonyms for CDDs (e.g. volunteer health worker, village health worker), the disease conditions (e.g. Onchocerciasis or river blindness), and for potential references to CDD motivation such as performance, retention, or satisfaction), with a highlight on such programs in Africa. This was important to identify studies reporting limiting and facilitating conditions affecting CDDs in NTD programs that may not have included the terms “incentives” and/or “motivation”.

### Study eligibility criteria

One reviewer scanned titles and two reviewers independently screened and selected abstracts of relevant citations using pre-defined inclusion criteria (See Appendix 1—Protocol for Literature Review on NTDs conducted in a systematic manner).

Inclusion was limited to English Language literature reviews and studies conducted from 1995 to September 2016 on MDA, school- or community-based drug distribution that involved the use of NTD volunteers in developing regions, and which assessed, described, investigated, or reported any factors affecting CDD motivation, performance, retention, or satisfaction. Studies were also included if they observed, analyzed, or documented any incentives for CDD involvement in NTD drug distribution programs, whether honorary or material, that provided an explanation for NTD volunteers motivation, performance, retention and satisfaction.

### Quality assessment of included studies

Quality assessment of included studies was performed using the Critical Appraisal Skills Program (CASP) qualitative research and systematic review checklists.

### Data collection and analysis

Two reviewers independently tested the data extraction form (Table 1) using a random sample of about 10% of the included studies. This process enabled necessary revision to establish reliability for independent data extraction. Full data extraction was then performed for all papers using a Microsoft Excel database. This included information on study design, country, NTD program, factors relevant to NTD volunteer participation, incentives and motivation, performance or satisfaction as described above. These data were then pooled and examined for themes. The themes identified included monetary and material incentives, intrinsic motivation, gender, cost to participate, health system and community support. Data were then re-categorized based on these themes and summarized.

**Table 1:** Example of data extraction table.

ID	Authors	Publication date	Countries	NTD Program	Study Design	Described or assessed factors associated with CDD motivation, performance, retention or satisfaction			
						Motivation	Performance	Retention	Satisfaction
**88**	Senyonjo L, Oye J, Bakajika D, Biholong B, Tekle A, Boakye D, Schmidt E, Elhassan E	2016	Cameroon	Onchocerciasis	Survey, focus group discussion	Supervision and support from the health facility workers did not appear to be a strong motivating factor. In fact, a number of CDDs expressed concern over a lack of support they received in handling potential adverse events and they often had to spend their own money to care for the patient with complications. The only positive incentive mentioned by CDDs was training. However, attending training often resulted in financial losses for CDDs, which also discouraged them from work.	The findings suggest that poor CDD motivation and mistrust between the community and the CDDs can be related to both a poor quality campaign and poor drug compliance.		Some CDDs felt that their work was not appreciated by the community, many experienced problems ranging from the community apathy and mistrust around CDDs’ motivations to negative reactions and insults in response to adverse side effects.

## Results

**Fig 1:**
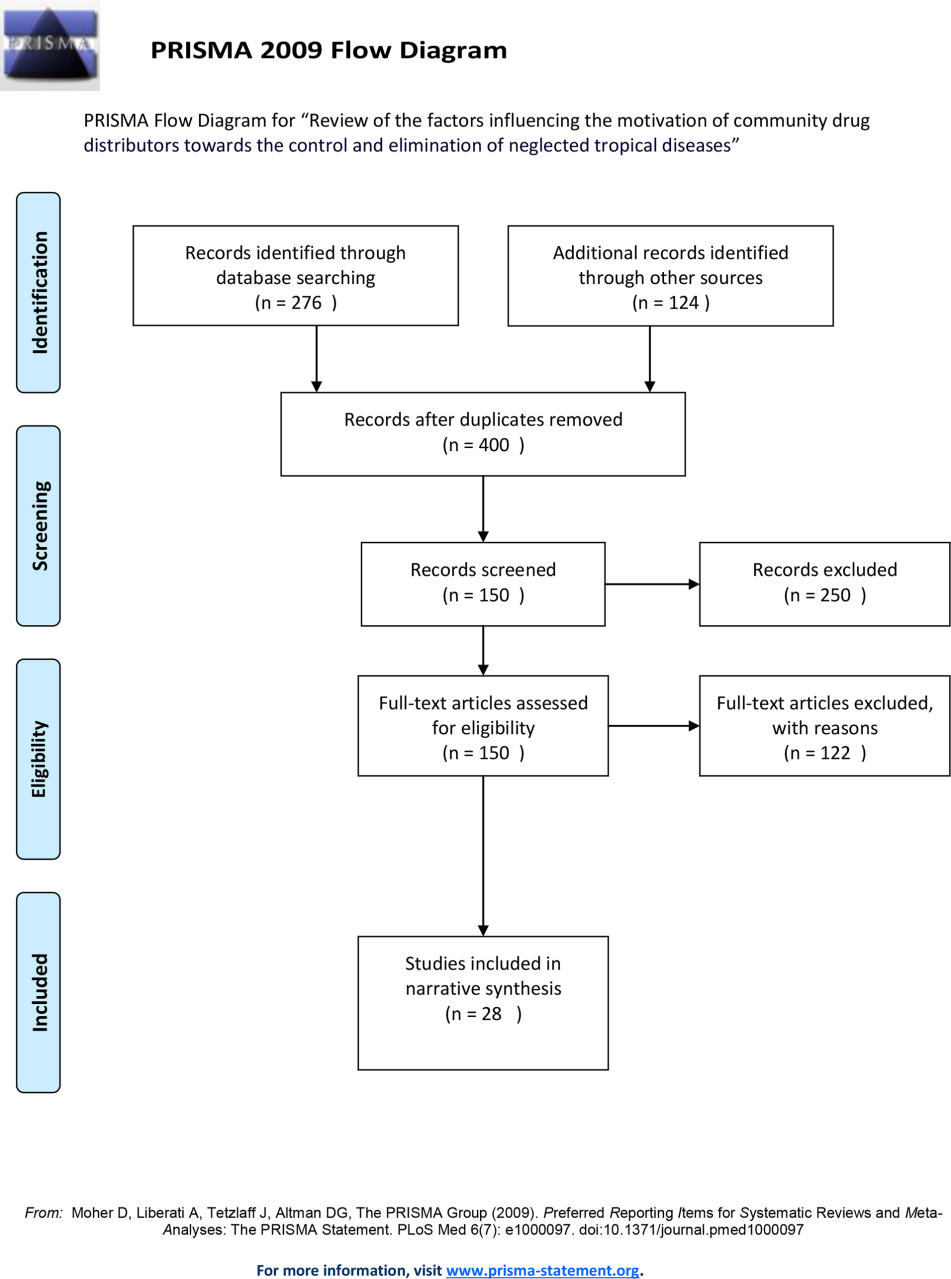
PRISMA flow diagram.

**Fig 2:**
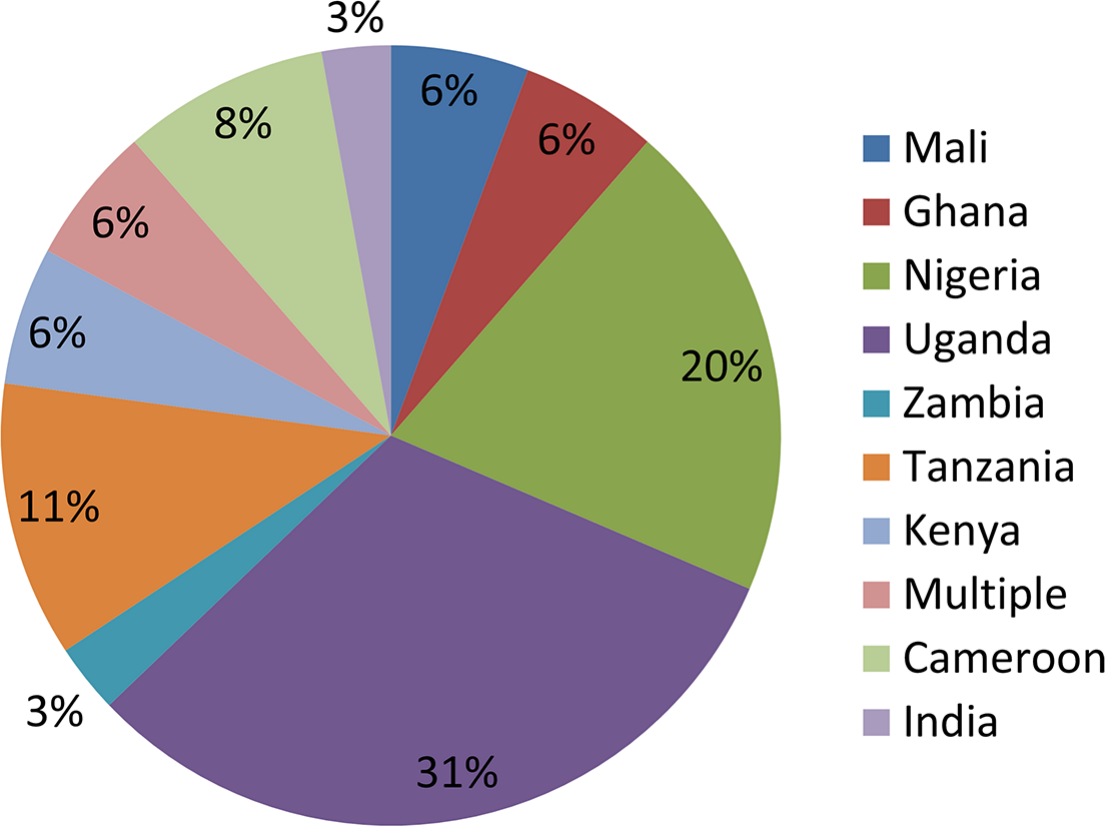
Percentage of publications by country.

**Fig 3:**
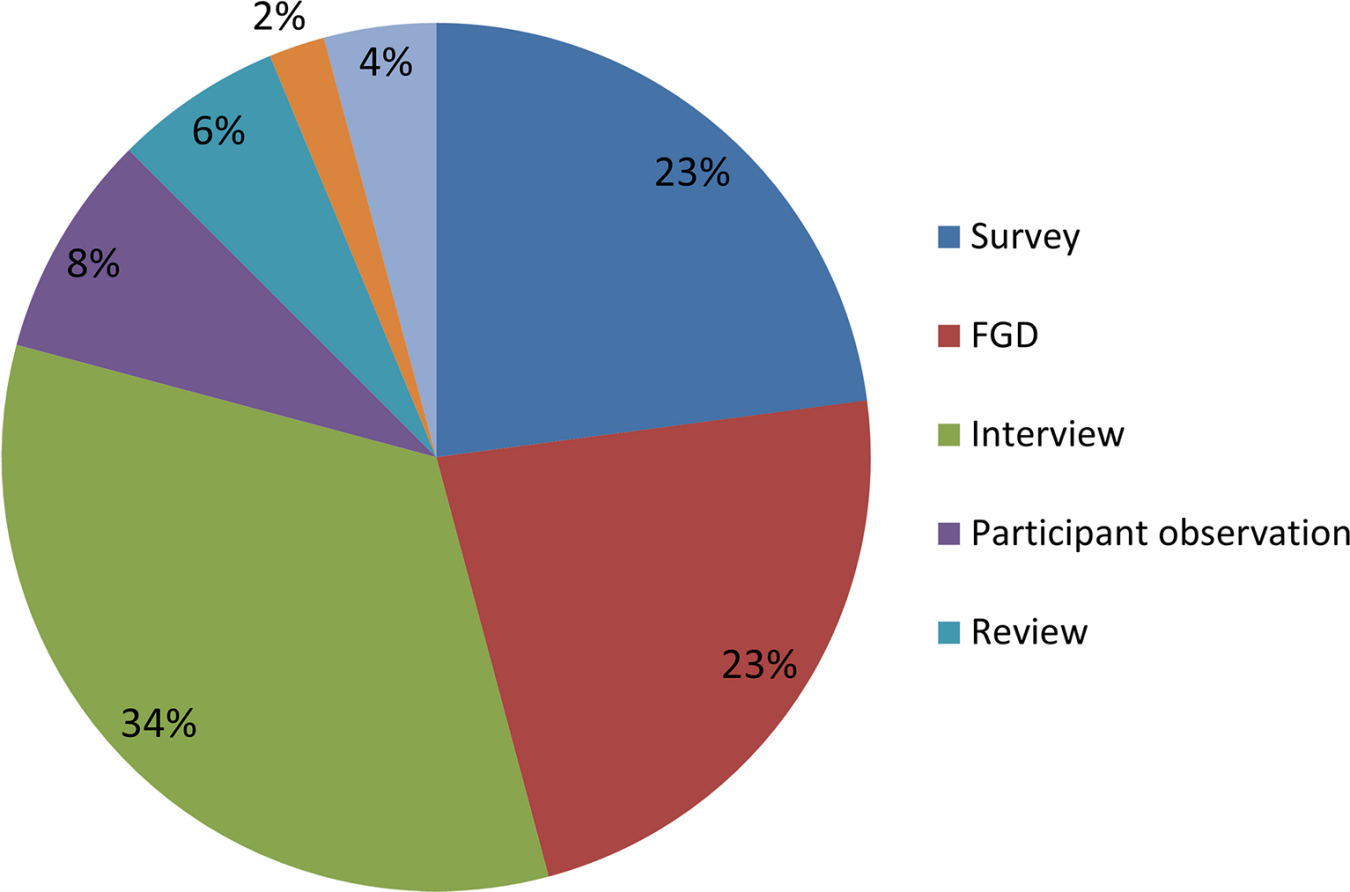
Percentage of publications by study design.

**Fig 4:**
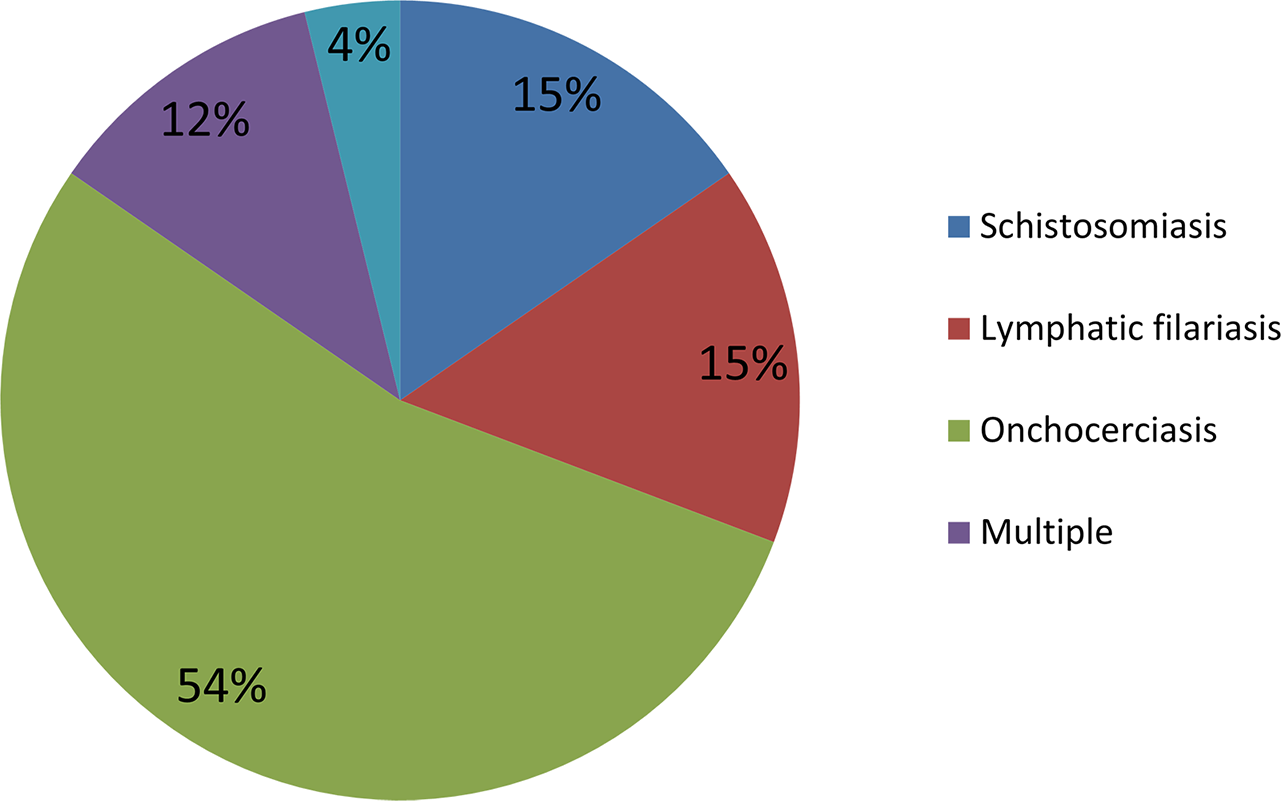
Percentage of publications by NTD.

A total of 28 studies were included in the final review (Fig 1). Most of the included studies were performed in Africa (Fig 2) with studies based in Uganda (31%) and Nigeria (20%) being the most common. Various study designs were represented; however, interviews (34%), focus group discussions (23%) and surveys (23%) were most common (Fig 3). Many studies employed mixed methods study designs. The majority of studies came from onchocerciasis (54%) programs, followed by lymphatic filariasis programs (16%, Fig 4).

The data from the papers can be grouped into the following thematic areas: monetary and material incentives, intrinsic motivation, gender, costs associated with participation as an NTD volunteer, health system factors (training, supervision, workload, timing, definition of roles) and finally community support. Each of these themes will be developed in more detail below.

### Monetary and material incentives

Material incentives were frequently cited in the literature. The same kinds of incentives were repeatedly mentioned in the examined studies. These were typically a stipend or items that would facilitate the NTD volunteers’ activities such as a bicycle, t-shirt, identity (ID) card or meals. A study by Fleming *et al* (2016) in Uganda found that 42% felt that a uniform was necessary so that they would be clearly identified as an NTD program implementer and a majority of NTD volunteers interviewed (67%) felt that a bicycle would facilitate their activities. Indeed, in a study by Halwindi *et al* in Zambia it was found that schistosomiasis control program volunteers were able to treat a higher proportion of children when they had access to a bicycle from the community at some times (p<0.001). Fleming *et al* (2016) also noted that t-shirts, bags, hats, boots and waterproof coats would also be considered as appropriate incentives.[15] Similar results were reported by Da Costa Vroom *et al* (2015) where volunteers indicated ID cards, t-shirts, hats, bicycles as well as preferential treatment at the district hospitals or health centers would be considered good compensation.[16] Nuwaha *et al* (2004) reported that in a Ugandan onchocerciasis program, the community might compensate volunteers with food or drink items or exempt them from community work. Some volunteers were also given money to buy lunch.[13]

NTD volunteers in the Fleming paper also mentioned that financial incentives should be given to compensate for opportunity costs incurred by volunteers during their NTD activities.[15] These findings were additionally reported by Katabarwa *et al* (2001) who wrote that onchocerciasis volunteers in Uganda requested free mid-day meals[17] and Brieger *et al* (2002) noted that low morale amongst volunteers in an onchocerciasis program was caused by lack of incentives to compensate for time spent on distribution.[18] Dabo *et al* (2013) also noted that schistosomiasis volunteers in Mali dropped out early as the work load was too heavy for the financial compensation offered by the program.[19] Dissatisfaction with material incentives provided was thought to originate from comparisons made by NTD volunteers between the NTD program and other health programs that also provided material or financial incentives.[20] However, a Nigerian study by Emukah *et al* (2008) found that only 5% of onchocerciasis volunteers paid by other health programs ceased volunteering (p<0.001) for the onchocerciasis program.[21]

Amongst the studies reviewed, none established a direct relationship between incentives and performance. Amazigo *et al* (2002) in fact found that in Cameroon and Uganda there was no significant association between giving monetary or in-kind incentives to onchocerciasis volunteers and treatment coverage (X^2^ = 1.089; P>0.05), demonstrating that providing incentives to volunteers did not necessarily improve treatment coverage. In the same study, treatment coverage was generally higher in communities where NTD volunteers were not given incentives versus those where they received financial incentives indicating that the willingness to continue service as an NTD volunteer was not statistically associated with the provision of financial incentives.[20] However, Nuwaha *et al* (2005) found that some CDDs cited non-payment of allowances as being one of the problems they encountered in their work. Some CDDs adopted the practice of charging a fee for tablets and this hindered compliance.[22]

### Intrinsic motivation

Although several studies have noted that various material incentives might improve NTD volunteer motivation, many studies found that the volunteer’s intrinsic motivation was a strong factor.[15,16,23–27] Some studies noted that NTD volunteers saw their activities as a way to serve the community.[15,16,23,24] Improvement in social status for volunteers was also an important motivating factor. [15,18,24,25] For example Fleming *et al* (2016) found that 61% of NTD volunteers interviewed considered recognition from the community as a doctor as one of the motivating factors for their work.[15] This was also noted by Sama *et al* (2003) who found that high self-esteem from status enhancement was a significant non-monetary incentive for onchocerciasis volunteers in Cameroon, Nigeria and Togo.[25] Brieger *et al* (2000) also noted that many volunteers were motivated, not by financial incentives, but rather by gains in “recognition, self-esteem and knowledge” (page 1).[28] Similar results were found by Adeneye *et al* (2007) in Nigeria who saw that schistosomiasis volunteers found their role as a way to serve their community and acquire an increased status in the community.[24]

Knowledge was found to be a key motivator for volunteers. [15,23,24] Fleming *et al* (2016) found that 36% of volunteers said they participated in the NTD program to increase knowledge on health issues.[15] Many volunteers in the Nigerian study by Mafe *et al* (2005) saw their participation as an opportunity to improve their knowledge on schistosomiasis.

Religious commitment was also shown to be an important motivation for some NTD volunteers. Senyonjo *et al* (2016) quote one NTD volunteer in West Cameroon as saying that even though they are not paid for their work, God will also help them out someday as a result. In Ghana an NTD volunteer commented that this work was “all part of God’s work.”[16]

Many NTD volunteers were also motivated by the trust placed in them by the community. In addition, they reported feelings of obligation towards the community and community expectations. A participant in the Fleming study (2016) said that he was motivated by the trust placed in him by the community.[15] Katabarwa *et al* (2001) noted that community pressure was a significant motivating factor.[27] Njomo *et al* (2012) described in a Kenyan study that more than one-fifth of the volunteers in the study agreed to distribute LF drugs because of the trust and recognition they received whereas one-fifth of the volunteers felt obliged since many people disliked volunteer work. [29]

### Gender

The effect of gender on NTD volunteer performance and motivation is currently an understudied area. Few included studies addressed the issue of gender explicitly however those that did found noteworthy results. [14,30–33] For example, Omedo *et al* (2012) examined the experiences and perspectives of NTD volunteers on the MDA program and showed that female volunteers in schitosomiasis programs in Kenya found it challenging to balance their work and family responsibilities. A few reported abuse at the hands of their husbands for coming home late and had to justify the benefit of being an NTD volunteer to them, e.g. that they would eventually be paid for their efforts. Some of the NTD volunteers said they continued with their work despite this, but this could be a de-motivating factor for others.[14] A paper by Parker *et al* (2011) in Uganda found that gender could also affect women’s performance as NTD volunteers due to existing social hierarchies. Participant observation of young female volunteers in schistosomiasis and soil-transmitted helminth distribution programs showed that it was impossible for them to insist that older men swallow their tablets in their presence, leaving them to take the tablet at a time of their choosing or not at all.[30] This result was also amongst the findings of O’ Connor *et al* (1999) whose study in Tanzania looked at characteristics of effective NTD volunteers and found that unmarried female volunteers had lower odds of good performance (OR: 0.22) as measured by the average outcome of children being treated for trachoma. This result was statistically significant (CI: 0.06–0.83).[31] Despite these setbacks, female NTD volunteers also have an advantage. As Brieger *et al* (2002) reported, the presence of female NTD volunteers can have a positive influence on coverage, noting, however, that more indepth study is required.[18]

Given the potential effects of gender on motivation and performance, gender can also affect attrition rates. A survey study with NTD volunteers performed by Katabarwa *et al* (2005) in Uganda found that one of the reasons provided by female volunteers for not continuing on in their work was getting married outside their kinship zone. After marriage it was expected that married female volunteers stay within the kinship of their residence so as to fend off malicious rumours regarding their integrity, effectively excluding them from work requiring them to travel outside their zones. [33] Some men do report being happy to see women as NTD volunteers, even their own wives.[17]

Female NTD volunteers have been shown to outperform their male counterparts in terms of distribution and professionalsm. A study by Katabarwa and colleagues found 67% of the female volunteers who were involved in onchocerciasis as well as other health activities had treated at least 90% of their ultimate treatment goal within a one-week period, as compared to 62% of male volunteers (p<0.05).[33] Also in Uganda, although female NTD volunteers may have lacked the experience of their male counterparts, they performed as well as the men did, and sometimes even better during distribution.[34] The female NTD volunteers in this study were better organized, e.g. allocating households among themselves fairly so that they could achieve ivermectin distribution within a few hours, completing their reporting jointly and sharing information on defaulters. [34] Another study showed that the overall perception was that women were as capable as men in distributing ivermectin. [17]And in the same study, of the 258 women interviewed in the community, 38% found women to be more patient and tolerant than men, 32% felt women were more committed than men and 28% agreed that although men were more active, women were more patient.[17,35]

The involvement of women in the planning, decision-making and implementation of CDTI was highlighted in 2002 as women represented only a small proportion of the NTD volunteer workforce.[32] Some of the reasons women have been less likely to be involved relate to local cultural expectations.[17,32] Being a woman within the prevailing cultural context can have implications on the female NTD volunteer’s relationship within the community. Katabarwa *et al* (2001) found that there were issues of trust amongst females in the Ugandan community since some of them married into the community and as such, came from outside. They reported that some women preferred not to have female NTD volunteers as these women believed that if they were to get into everyday quarrels with their female volunteer neighbours, they might abuse their power and withdraw services. It seems within this Ugandan context that men have more opportunities to resolve their quarrels whereas the women lack an established structure to resolve their disputes, allowing them to become grudges over time.[17] This cultural context can also be advantageous for female NTD volunteers. For example, they were considered to be more reliable than the male volunteers because they only served within their kinship zones.[33]

### Cost to participate

Opportunity costs are known to be high for NTD volunteers since they have to spend time on program activities at a loss to their own personal responsibilities. A qualitative study of those involved with the MDA program for LF by Da Costa Vroom *et al* (2015) found that in Ghana, the timing of the program coincided with cocoa harvesting and the small-scale gold mining period, both which pay better than working in the MDA. As such, many NTD volunteers were inclined to leave the program in order to join this activity instead.[16] Another source of opportunity costs came from NTD volunteers having to forego their own household duties to perform their work as noted by Parker *et al* (2011).[30] Participants in a study by Fleming *et al* (2016) mentioned that they carry out their NTD activities for free, however the household costs for food and school fees remain in place.[15]

In addition to the loss of revenue, NTD volunteer work can also result in out-of-pocket expenses. In a Ugandan study, the out-of-pocket expenses for NTD volunteers ranged from 1.32–8.61 USD per person averaging at 2.78 USD. These costs were mostly for transportation and lunch.[15] Out of pocket costs for transportation paid by NTD volunteers were also reported in an onchocerciasis program in Nigeria.[18] In addition to these costs, NTD volunteers have also paid out of pocket for materials needed to carry out the NTD program. A study by Brieger *et al* (2000) in Nigeria, Ghana, Mali and Togo found that some communities were not contributing towards the costs of buying necessary supplies thus leaving the NTD volunteers to spend their own money.[28] Another cost incurred by NTD volunteers was associated with attending training sessions. Research by Senyonjo *et al* (2016) in Cameroon showed that this discouraged people from NTD volunteer work. One participant lamented that he had to pay for transport to attend volunteer meetings without being reimbursed and for this reason, he decided to resign.[36] Finally, in this same study, some NTD volunteers covered the costs to assist people who had adverse events as a result of the NTD treatment since little or no support was available from the health facility.[36]

### Health system

Various health system issues were found to be either a hindrance or an advantage for NTD volunteer motivation and performance. A frequently occurring theme was the impact of proper training. [20,26,36–38] Senyonjo *et al* (2016) found that in Cameroon, NTD volunteers cited training as a positive incentive however as mentioned above, the cost to attend was problematic for some. When proper training was not received, authors in several studies found that NTD volunteers were unable to perform their duties well in terms of educating participants or dealing with questions that arose during the distribution.[10,20,26,37,38] Onchocerciasis volunteers in the Nuwaha *et al* (2004) study in Uganda reported that inadequate training was the cause of their inability to explain the disease properly.[13] Improper training hindered the performance of the program as seen by Hussain *et al* (2014)[39] and had the additional effect of creating mistrust of the community towards NTD volunteers and the program in Uganda.[37] A study examining factors affecting community participation in a Tanzanian onchocerciasis program by York *et al* (2014) noted that there was a lack of education seen in both the community members and the NTD volunteers.[10] Similarly, Fleming *et al* (2016) suggested that factors contributing to poorer volunteer performance could be decreasing the amount of time recorded on health education and mobilization activities.[15]

Another issue negatively affecting NTD volunteer performance was associated with supplies provided through the health service. One study found that drug stock outs and issues around drug packaging resulted in poor NTD volunteer performance and lower coverage.[15] A study assessing predictors of compliance by Nuwaha *et al* (2005) in Uganda found that the lack of facilities for proper storage and dispensing of tablets hindered the NTD volunteers’ work.[12] Hussain *et al* (2014) reported that non-availability of drugs to treat side-effects in India made it difficult for volunteers to carry out their tasks.[39]

Supervision by health staff of the NTD volunteers has been shown in some studies to have a direct impact on their performance. Dabo *et al* (2013) found that higher coverage rates were achieved when formal health workers were present at the time of distribution (p<0.01).[19] Conversely, Senyonjo *et al* (2016) found that lack of support received from health staff for handling potential adverse events was a de-motivating factor for NTD volunteers.[36] These results were corroborated by Njomo *et al* (2012) who found that lack of supervision from health workers could reduce volunteer motivation in a Kenyan program.[29] Similarly, Hussain *et al* (2014) reported that lack of follow-up by volunteers and health workers, delays in receiving funding and supervisors not fulfilling their tasks adequately hindered the functioning of the program.[39]

The heavy workload expected of NTD volunteers can both positively and negatively affect their motivation. In a Malian study looking at schistosomiasis control, the researchers reported that allocation of too many households to NTD volunteers negatively affected their performance and motivation, as they found themselves overburdened with work.[26] Rilkoff (2013) found analogous results in Uganda.[37] Similarly Kisoka *et al* (2016) found through interviews in Tanzania that NTD volunteers thought the program did not provide enough time for them to visit all their allocated houses and those that continued the work after the time limit were not compensated. Volunteers in India reported a similar issue in a study by Hussain *et al* (2014). They reported that it was difficult to distribute all the medicine in a single day.[39] In Ghana, NTD volunteers indicated that they needed a longer timeframe for drug distribution so that they could achieve a higher coverage for MDA.[16]

Katabarwa *et al* (2005) found somewhat contrasting results that suggested adding more responsibilities to NTD volunteers actually enhanced their performance, and did not result in an increased attrition rate.[33] Fleming similarly found that being involved in multiple activities was perceived by volunteers to improve their performance and make it easier to mobilize the community for the NTD program, however spending more time on NTD program activities decreased coverage. [15]

The timing of the MDA may also be challenging for performance of NTD volunteers and the programs. In a study by Kisoka *et al* (2016), MDA for LF occurred during the rainy season making it difficult for volunteers to reach all the houses. [38] Omedo *et al* (2012) found that in Kenya, volunteers in the schistosomiasis control program reported it was difficult to convince people to take the drugs as they did not have enough to eat, exacerbating side effects. As a result, villagers suggested having the distribution during the harvest time when there is enough food.[14] In the same Kenyan study, the NTD volunteers reported that the distribution was scheduled at the time when the community members were tending to their farming plots, making it harder to find people at home. Similarly, as mentioned above, MDA for LF in Ghana occurred at the same time as cocoa harvesting season, resulting in many volunteers opting out of the program and in the prolongation of the distribution period.[16] It is interesting to note that none of the papers that looked at onchocerciasis programs mentioned issues with the timing of the MDA. This may be a reflection of the the CDTI approach, which these programs follow, that gives communities autonomy in program planning, allowing for them to choose times that are in tune with their respective community calendars.

Another potential source of low performance in NTD volunteers may be lack of clarity in allocation of the MDA roles. The Omedo (2012) study on schistosomiasis control in Kenya found that there was some confusion between those responsible for the census/sample collection and those distributing the drugs that resulted in community members being told not to take the treatment.[14] The lack of consensus within the village about the roles, responsibilities and motivations of APOC volunteers was associated with volunteers’ demands for incentives as noted by Amazigo *et al* (2002).[32]

### Community support

An overwhelming number of studies pointed to various aspects of community support as being related to both NTD volunteer motivation and performance. Noted by both Amazigo *et al* (2002) and Brieger *et* al (2000), NTD volunteers felt de-motivated when communities did not contribute towards purchasing supplies or helping with activities necessary to perform the work.[20,28] The converse is also true, when communities were actively involved in the MDA, volunteers reported improved motivation. A study by Njomo *et al* (2012) in Kenya found that “positive peer influence through assistance in drug distribution and community sensitization contributed to volunteers’ motivation”. [29] Emukah *et al* (2008) similarly reported that volunteers who were assisted in tablet collection by the community were more likely to distribute their drugs quicker.[21] Halwindi *et al* (2015) found in their assessment of a community-directed treatment for STH in Zambia that “a higher proportion of children were treated by volunteers who received assistance in mobilizing community members” compared to those who received no assistance (p = 0.006).[40] Dabo *et al* (2013) found that in high coverage villages it was common for the community to assist in health education, mobilization and obtaining census materials.[19] Brieger *et al* (2002) found similar results in onchocerciasis control in Nigeria noting that in low coverage villages, the community did not help and did not cooperate.[18]

Lack of community appreciation and respect for volunteer efforts was also found by several studies to result in low motivation and poor volunteer retention.[25,27,28,36] A study by Katabarwa (2016) found that 96% of volunteers thought that the community did not appreciate their efforts and 93% were not willing to continue as a volunteers.[27] Similar results were found in Cameroon by Senyonjo *et al* (2016) with volunteers working in the onchocerciasis program who also reported that they were not being appreciated by the community. One CDD reported, “If the community valued me then at least one father or mother would have called me someday and thanked me simply. Just simple gratitude.”[36] Sama *et al*. (2003) found that when communities appreciated the work of volunteers, it motivated them.[25] Fleming *et al* (2016) showed that volunteers did not agree that the community support was sufficient to motivate them. The majority mentioned that further support was needed from the Ministry of Health and mentioned various material incentives. [15]

Other community related factors included the community response to adverse drug reactions and their respect for the role of the volunteer. In studies by Brieger (2000) and Senyonjo *et al* (2016), it was reported that if people in the community developed side effects they lashed out at distributors.[28,36] Community members were also reported as not respecting the concept of a volunteer. Brieger (2000) found that community members did not respect the volunteers’ time, approaching them at any time of the day. Furthermore, they were often absent during distribution, requiring the volunteer to visit again.[28]

## Discussion

The indispensable role of the NTD volunteer to the success of control and elimination goals is certain. Through their work globally, these volunteers have brought life-saving and health-improving care into the homes of billions of people for decades. For these reasons it is critically important that we understand how these NTD volunteers are presently motivated and how their performance can be sustained and improved, particularly as the timeline of 2020 approaches. This review compiles the literature specific to the motivation and performance of the NTD volunteers across five PC diseases and offers some insight into the key thematic areas affecting motivation and performance.

NTD programs are not unique in their use of volunteers for community outreach and service delivery. Like the community health worker (CHW), the NTD volunteer is not always remunerated for his/her work, is not a formal part of the health system and is often the direct link between the health program and the community. In many instances, NTD volunteers are CHWs, carrying out other health activities in the community. This review highlights some of the challenges that face volunteers in their work in NTD control and elimination. The interaction between the involvement of the volunteer in community health activities, other than NTD activities, was not a predominant theme that emerged in this review. Many of the thematic areas identified in this review echo other findings from research focusing on community and lay health workers.

Intrinsic motivations, for example, are not unique to NTD volunteers and have been shown in CHWs as well. A Cochrane qualitative evidence synthesis described how lay health workers from low, middle and high income countries were motivated by altruism, social recognition and improved knowledge. The authors also highlighted the contradiction that exists between lay workers’ views on incentives: some wanted regular payment while others were happy to volunteer without financial incentive.[41] Kane *et al* (2016) in their review of CHW empowerment in six countries reported on some of the positive intrinsic motivations of CHWs like being called a doctor and being trusted by the community. They also mentioned the value of improved competency, particularly for curative tasks. [42] Given the consistency of intrinsic motivation as a theme across various NTD studies, programs would benefit from identifying ways to reinforce these values. One suggested method is to increase community appreciation for NTD volunteers. Community members should understand that volunteers are not receiving a salary for their service, hopefully increasing assistance for volunteers as well as gratitude. This may be especially relevant in some communities where individuals believed that volunteers were being paid.[28] Another suggestion is to institute ways for the health service and community to publicly recognize the NTD volunteers’ work and contribution.

Studies from other fields also describe factors related to low motivation of health volunteers that are similar to what has been described in the NTD literature. A study of health promotion volunteers in Japan found that volunteers with low motivation also reported uncooperative neighbourhood associations.[43] Another demotivating effect for CHW was when materials were not available for fieldwork and when supervision focused only on reporting forms. [42]

The literature on motivations, incentives and performance of community and lay health workers is more substantial to what has been documented about NTD volunteers. One of the key differences between the NTD literature and the general health literature is the limited amount of evidence of the impact of community or lay health workers on the health of the populations they serve. For certain areas, like pneumonia and diarrhea, there was little evidence of CHW impact. [44] However, a larger impact has been demonstrated where CHWs were engaged in delivering insecticide treated nets and malarial chemoprophylaxis as shown in a systematic review from sub-Saharan Africa. [45] In NTD programs, the NTD volunteers have a clear impact on programmatic outcome, as they provide the most efficient mode in which to deliver drugs to the community.

One of the areas identified in this review is the impact of the health system on the NTD volunteer. The health system, usually at the district level with the support of frontline health facilities, is responsible for training, supervision and providing the material support for NTD volunteers so that they can perform their activities in the community. The health systems in the countries where NTDs are endemic are often constrained by insufficient funding, limited human resources, insufficient management and poor governance.

Evidence shows that vertical disease control programs can further strain already weakened health systems, [46–48] suggesting that NTD programs have not played a strong role in strengthening health systems. NTD volunteers experience health systems challenges in the execution of their duties. For example, when training is inadequate to meet the needs of volunteers, support for adverse events is insufficient or when supplies do not arrive in time for program activities, the challenges of the health system become the challenges of the NTD volunteer. Therefore, identification of solutions to these issues requires a systems approach, something that global health initiatives, like NTD control and elimination, have not always considered and have sometimes even resisted. The way forward to sustain and improve motivation of NTD volunteers must be accomplished within the framework of health systems. NTD programs provide excellent opportunities for health systems strengthening,[49] however it is rarely the expected output of program activities.

Better integration of NTD programs into health systems is seen as a critical way to ensure sustainability of the gains made in the NTD elimination program as well as to improve impact of elimination activities by integrating the NTD program into existing activities. Integration in the health sector has been variously defined and applied to achieve ends broadly conceptualized as reducing fragmentation and duplication of interventions to achieve a continuum of health care, ease of access, improved health outcomes, wide population coverage, user satisfaction and efficiency. [50–52] The one-stop shop health care service, the delivery of multiple population based interventions on a single platform for efficiency and improved population coverage and a process where disease control activities are functionally merged or tightly coordinated with multifunctional health care delivery are all forms of integration. [53–58] In many countries, it is at the lowest level of program implementation that the most integration occurs especially if the service providers have the flexibility of managing their own resources. In a study by Mensah et al (2016) on the level of integration of the NTD program into the regular health system, the authors indicate that the level of integration was much higher at the district level compared to the national and regional levels.[59] This was because the District Health Management team (DHMT) operated an integrated unit coordinated by the District Director of Health Service.

Although there has been an increase in the number of countries integrating community-based health workers, including the NTD volunteer, into the health systems, a systematic review on the integration process of national community-based health worker programs into health systems in low and middle income countries showed that integration has not been optimal. This is because integration has been influenced by levels of trust, appreciation and support by community and family members as well as the attitudes of professional health workers.[41,60] For sustained success of NTD programs, there is a need to critically look at proper integration of community health workers, like NTD volunteers, into public health programs at the lowest level of health care delivery as well as encourage a national priority to ensure efficiency and better delivery of health services. The NTD volunteer workforce has enormous potential for other public health activities. This should encourage the process of better integration of this workforce into the health system to strengthen the delivery of public health initiatives at the community level.

An additional recommendation to reduce the added pressure on health systems is to ensure better coordination between global health initatives to allow for better synergy between horizontal and vertical programming.[61] This coordination ideally should occur at all levels, from the level of global donors through to the actors at the community level. We know that integration at the national level is largely dependent on the strength of the health system itself to deliver its programs and as such, integration may be more feasible in some national contexts as opposed to others. [62] A functioning integrated system is expected to increase efficiency of program delivery and has the potential to aid NTD volunteers in their work by improving supervision, clarifying reports as well as ultimately decreasing the amount of time they are required to spend away from their other household responsibilities. This may also improve CDD satisfaction by reducing comparisons between programs. Differences in donor-directed payment of incentives has been shown to cause dissatisfaction; this is particularly relevant since geographical overlap of programs is quite high.[63]

Another challenge that has been highlighted in NTD programs is the multiple years that volunteers have been engaged in program activities. Many MDA programs for LF have progressed to more than six rounds, and for onchocerciasis, more than 10 years, creating fatigue for the health providers, NTD volunteers and the communities at large. This prolonged use of volunteers has had the benefit of mobilizing and training thousands of people for health. However because the end date of many of these MDA programs is unknown, volunteers express weariness and discouragement as their tasks continue on year after year. In addition, for the NTD volunteer who started with once yearly MDA for LF or onchocerciasis, his or her tasks now comprise a suite of activities including drug delivery for multiple diseases at different times of the year. The literature on this is mixed, as seen in those papers citing that volunteers feel overwhelmed by the workload and the short time they have to complete their duties, while other researchers have reported that the increase in workload has had a beneficial impact on the NTD outcomes. Better understanding is needed on the impact of this programmatic transition for the volunteer and on how to more effectively introduce changes to their training and routine tasks.

One of the important findings in this review is the significance of community support on the motivation and performance of the NTD volunteer. Volunteers come from the communities that they serve and the literature supports the need for these communities to be engaged and cooperative when the volunteers carry out their work. This means ensuring that communities understand the importance of the goals and benefits of the program, that they are engaged with the efforts and that they are part of program implementation especially when and where distribution will occur. When the support of the community is not present, the effect can be demotivating for the volunteers. As the NTD programs in many countries progress, the community engagement process should also evolve sufficiently to support elimination efforts. Communities should know program successes and understand program planning with regards to transmission assessments and stopping MDA. In communities where MDA programs have existed for many years, people no longer see disease in the same way that they did at the start of these programs, thus lessening community perception of risk. New messages will help communities to see their successes, but also recognize the need for sustained support until transmission is interrupted. Good community engagement practices can impact directly the work of the volunteer, providing the reception and affirmation he or she needs to carry out drug distribution, referrals for adverse events, and reporting. Any solution to improve and sustain the motivation of the volunteer must take into account improved community engagement, particularly in the “older” campaigns. This could be done by involving the community in reaching targets by allowing them to identify appropriate CDDs, testing MDA messages and involving key community leaders and existing groups in the MDA program. The hypothesis being that engaged communities should enhance volunteerism. Recent research by Chami et al[64,65] underlines the power of understanding and utilizing social networks in relation to CDDs in MDA planning and implementation. It was shown that people with many social connections and in a central location were more likely to be offered the MDA. In another paper, Chami found that certain groups of people may be less likely to be approached during the distribution.[66] By combining knowledge of non-recipients and social networks, villages may be able to identify CDDs who would be able to reach a greater proportion of the population.

The review has highlighted some of the out of pocket expenditures that volunteers have in their activities. While the average cost per NTD treatment has been reviewed recently (Fitzpatrick et al 2016), the authors note that few cost studies included in the systematic review and model provided information on the opportunity cost incurred by volunteers or the out of pocket expenses that some volunteers incur. The cost of $0.50 per treatment that is often used in NTD advocacy does not include the cost of volunteers. Fleming *et al* (2016) provide one of the only recent studies that have addressed volunteer costs. These costs, both economic and financial, must be considered in order for the NTD programs to not burden the volunteers and communities with unsustainable costs, particularly as the intensity of the end game for NTDs becomes more salient.

Increasingly, the impact of gender on health has been highlighted as crucial in our ability to understand the local context. This review has demonstrated that the experiences and performance of male and female volunteers are not the same. Female volunteers have been shown to be patient and conscientious; however there can be limitations on their ability to work due to household responsibilities and cultural constraints. Better understanding of these differences would help national programs to equip their NTD volunteers more effectively, knowing that the constraints and opportunities may be different according to the context. For example, how could female NTD volunteers be better supported in their work in the field? How can some of the skills attributed to female NTD volunteers be incorporated into the training of all NTD volunteers?

One of the limitations of this review is that most of the included papers center their research on the African context. This underscores the history of the CDD, which is rooted in the CDTI approach which was introduced and supported by APOC. In addition, it may reflect the use of more primary health care personnel in drug distribution in other regions as compared to community volunteers. [67] However, it also suggests that there is a dearth of research into the motivation and performance of NTD volunteers from other countries where these programs are ongoing and still expanding, namely South Asia, Southeast Asia and the Western Pacific regions. Another challenge is the limited evidence of the impact of gender on the volunteer. Although some papers do acknowledge gender in their reviews, it is not systematically included in the assessment of volunteer performance and motivation. Perhaps with a renewed interest in gender and NTDs, future research will provide a gendered approach in their analysis of these issues.

## Conclusion

This review has highlighted the need for more research on the role of incentives in motivation, retention and performance of community volunteers. The literature on volunteer motivation and performance is minimal, particularly outside of the African context and with regards to gender. Some of the literature reviewed demonstrates the link between improved drug coverage and some of the thematic areas covered in this paper, but these links tend to be anecdotal and limited by geographical boundaries. To show a definitive association between the introduction of a certain incentive or training program for NTD volunteers and improved drug coverage would be challenging due to the constraints of conducting research in non-controlled environment. As such, there is a need for more implementation research to demonstrate the utility, acceptability and feasibility of measures to improve NTD volunteer motivation. Any initiatives to improve and sustain motivation of NTD volunteers must be considered within the lens of the health system and changing sociocultural environment. This allows for a clear understanding of the local context and the possibility to adapt the training, supervision and incentivization of volunteers. Furthermore the gains of the NTD program can be sustained for use in other public health programs.

The use of NTD volunteers to date has appeared “free” until recent research elucidating their opportunity costs.[15] The NTD community needs a better understanding of the true costs that these volunteers bear by virtue of participation in the program, including the out of pocket expenses. Without a full understanding of these costs, the real value of the volunteer will be underestimated.

Finally there is an urgent need to identify effective resources and novel approaches to better equip NTD volunteers. The NTD program can learn from other health programs in the use of mobile phone technology, radio and theatre, for example, to reflect changes in the ways that communities receive information in a changing socio-cultural environment. As 2020 approaches, it is clear that the goals will not be met without the full support and cooperation of this NTD volunteer workforce.

## Supporting information

S1 Supporting Information List of papers accepted for review list of papers accepted for review in "Review of the factors influencing the motivation of community drug distributors towards the control and elimination of neglected tropical diseases (NTDs)". (PDF)
