# Supplementary material for: Review of the factors influencing the motivation of community drug distributors towards the control and elimination of neglected tropical diseases (NTDs)
Source: PLoS Negl Trop Dis. 2017 Dec 6;11(12):e0006065. doi: 10.1371/journal.pntd.0006065 (PMC5718409; doi:10.1371/journal.pntd.0006065)
Supplement: S1 Supporting Information — (PDF) [file pntd.0006065.s001.pdf]

S1 Supporting Information: List of papers accepted for review in "Review of the factors influencing the motivation of community drug distributors towards the control and elimination of neglected tropical diseases (NTDs)"

No	Title	Authors	Year	Countries
1	Sociocultural aspects of mass delivery of praziquantel in schistosomiasis control: The Abeokuta experience	Adeneye AK, Akinwale OP, Idowu ET et al.	2007	Nigeria
2	Monitoring community-directed treatment programmes for sustainability: lessons from the African Programme for Onchocerciasis Control (APOC)	Amazigo UV, Obono OM, Dadzie KY et al	2002	Uganda, Nigeria, Cameroon, Tanzania
3	The challenges of community-directed treatment with ivermectin (CDTI) within the African Programme for Onchocerciasis Control (APOC)	Amazigo UV, Brieger WR, Katabarwa M et al.	2002	Multiple
4	Factors associated with coverage in community-directed treatment with ivermectin for onchocerciasis control in Oyo State, Nigeria	Brieger WR, Otusamya SA, Oke GA et al.	2002	Nigeria
5	Implementation and sustainability of Community-Directed Treatment of Onchocerciasis with ivermectin: a multicountry study	Edited by Brieger WR	2000	Nigeria, Ghana, Mali and Togo
6	Data reporting constraints for the lymphatic filariasis mass drug administration activities in two districts in Ghana: A qualitative study	da-Costa Vroom FB Aryeetey R, Boateng R et al.	2015	Ghana
7	Factors associated with coverage of praziquantel for schistosomiasis control in the community-direct intervention (CDI) approach in Mali (West Africa)	Dabo A, Bary B, Kouriba B et al.	2013	Mali

8	Factors affecting the attrition of community-directed distributors of ivermectin, in an onchocerciasis-control programme in the Imo and Abia states of south–eastern Nigeria	Emukah EC, Enyinnaya U, Olaniran NS et al.	2008	Nigeria
9	A mixed methods approach to evaluating community drug distributor performance in the control of neglected tropical diseases	Fleming FM, Matovu F, Hansen KS and Webster JP	2016	Uganda
10	Re-assessing community-directed treatment: evidence from Mazabuka District, Zambia	Halwindi H, Magnussen P, Siziya S, Meyrowitsch DW and Olsen A	2015	Zambia
11	Mass drug administration for lymphatic filariasis elimination in a coastal state of India: a study on barriers to coverage and compliance	Hussain MA, Sitha AK, Swain S, Kadam S and Pati S	2014	India
12	Involvement and performance of women in community-directed treatment with ivermectin for onchocerciasis control in Rukungiri District, Uganda	Katabarwa MN Habomugisha P and Agunyo S	2002	Uganda
13	Involvement of women in community-directed treatment with ivermectin for the control of onchocerciasis in Rukungiri district, Uganda: A knowledge, attitude and practice study	Katabarwa M, Habomugisha P, Ndyomugyenyi R and Agunyo S	2001	Uganda
14	Community-directed interventions strategy enhances efficient and effective integration of health care delivery and development activities in rural disadvantaged communities of Uganda	Katabarwa MN, Habomugisha P, Richards Jr FO and Hopkins D	2005	Uganda
15	Community-directed health (CDH) workers enhance the performance and sustainability of CDH programmes: experience from ivermectin distribution in Uganda	Katabarwa MN and Richards Jr FO	2001	Uganda
16	Dilemmas of community-directed mass drug administration for lymphatic filariasis control: a qualitative study from urban and rural Tanzania	Kisoka W, Mushi D, Meyrowitsch DW et al.	2016	Tanzania

17	Effectiveness of different approaches to mass delivery of praziquantel among school-aged children in rural communities in Nigeria	Mafe MA, Appelt B, Adewale B et al.	2005	Nigeria
18	The Role of Personal Opinions and Experiences in Compliance with Mass Drug Administration for Lymphatic Filariasis Elimination in Kenya	Njomo DW, Amuyunzu-Nyamongo M, Magambo JK and Njenga SM	2012	Kenya
19	Predictors of compliance with community-directed ivermectin treatment in Uganda: quantitative results	Nuwaha F, Okware J and Ndyomugyenyi R	2005	Uganda
20	Predictors for compliance with community directed ivermectin treatment in Bushenyi District of Uganda: qualitative results	Nuwaha F, Okware J and Ndyomugyenyi R	2004	Uganda
21	Characteristics of effective Village Treatment Assistants: the Kongwa Trachoma Project	O'Connor J, Lynch M, Vitale S and West S	1999	Tanzania
22	Community Health Workers' Experiences and Perspectives on Mass Drug Administration for Schistosomiasis Control in Western Kenya: The SCORE Project	Omedo MO, Matey EJ, Awiti A et al.	2012	Kenya
23	Does mass drug administration for the integrated treatment of neglected tropical diseases really work? Assessing evidence for the control of schistosomiasis and soil-transmitted helminths in Uganda	Parker M and Allen T	2011	Uganda
24	Exploring Gender Dimensions of Treatment Programmes for Neglected Tropical Diseases in Uganda	Rilkoff H, Tukahebwa EM, Fleming FM, Leslie J and Cole DC	2013	Uganda
25	A multi-centre study of community-directed ivermectin distributors' (CDDs') involvement in other healthcare and development programme activities in Cameroon, Togo, Sudan, Nigeria and Uganda	Sama MT, Homeida M, Ngang P et al.	2003	Cameroon, Togo, Sudan, Nigeria and Uganda
26	Factors Associated with Ivermectin Non-Compliance and Its Potential Role in Sustaining *Onchocerca volvulus* Transmission in the West	Senyonjo L, Oye J, Bakajika D et al.	2016	Cameroon

	Region of Cameroon			
27	Contribution and performance of female Community-Directed Distributors in the treatment of onchocerciasis with Ivermectin in Sub-Saharan Africa: a systematic review	Vouking MZ, Tamo VC and Tadenfok CN	2015	Multiple
28	Factors Affecting Community Participation in the CDTI Program in Morogoro, Tanzania	York KJ, Kabole I, Mrisho M et al.	2014	Tanzania
